# A scoping review of mental health problems and associated psychological factors pertinent to young people in education in low-and middle-income countries

**DOI:** 10.3389/fpubh.2026.1842137

**Published:** 2026-06-18

**Authors:** Tara Linda Murphy, Abbie Smith, Cris Glazebrook, Elena Nixon

**Affiliations:** 1 Mental Health and Clinical Neurosciences, School of Medicine, Institute of Mental Health, University of Nottingham, Nottingham, United Kingdom; 2 Tic Service, Psychological and Mental Health Services, Great Ormond Street Hospital NHS Foundation Trust, London, United Kingdom

**Keywords:** LMIC, mental health problems, psychological factors, resilience, education-attending young people, self-esteem, youth mental health

## Abstract

**Introduction:**

The objective of this scoping review is to identify how adolescent mental health is assessed and the psychological factors it has been associated with young people in education in Low- and Middle-Income Countries (LMICs).

**Method:**

A review of the published academic literature was conducted to explore the breadth, scope, and nature of research using the eligibility criteria of school/college-attending young people (aged 4–22 years) from LMICs with use of standardized measures. Three databases were searched: Medline, EMBASE, and PsycINFO, covering the period from 1970 to September 30, 2025. A logic grid using relevant population, construct, and context search terms guided the search. The six-stage scoping review methodological framework by Arksey and O’Malley was used.

**Results:**

The search yielded 1,493 records, of which 498 duplicates were removed and 995 were screened. Thirty six studies met the inclusion criteria. Thirty-two of these studies were conducted in middle-income countries. Ten of the articles described intervention studies and most of the studies were cross sectional. Mental health outcomes assessed were depression, anxiety, trauma, suicidality, eating disorder symptoms, psychological distress and general mental health screening. Assessed psychological factors associated with mental health outcomes included self-esteem, self-efficacy, resilience, coping, perceived social support, school connectedness, emotional intelligence and emotional regulation, mastery, and life satisfaction. Findings show that depression was the most commonly assessed mental health outcome and self-esteem was the most frequently researched psychological factor.

**Conclusion:**

The study revealed considerable variability in how mental health was assessed, underscoring the need for greater consistency and contextual sensitivity. These findings highlight the importance of developing interventions tailored to local contexts in LMICs to more effectively support youth mental health. The need for measurement consistency and contextual adaptation of intervention in this literature is indicated.

## 1 Introduction

Low- and Middle-Income Countries (LMICs) have younger populations than High Income countries (HICs) and mental health problems such as depression and anxiety, are common in these nations (1). Young people, a term used in this paper to describe children, adolescents and young adults, living in LMICs often face compounded risks to mental health and psychological development such as poverty and educational disruption (138) and exposure to violence (2, 3). Despite such reported issues in LMICs, research and interventions for mental health problems have historically been conducted in or informed by HICs (4). However, a focus on young people, which spans the full breath of youth is warranted, particularly in light of the literature suggesting the increasing vulnerability of their mental health as responsibility increases toward adulthood and institutional support potentially reduces (5, 6).

Recent studies show the multidimensional nature of youth mental health in LMICs, highlighting the need to improve it through psychological interventions and other means of well-being support, including school-based mental health promotion and intervention (7–10). However, the current interventions can lack cultural or contextual adaptation and comprehensive evaluation, limiting their scalability and sustainability (4).

Much of the existing literature of mental health problems in young people living in LMIC has been carried out in response to war or natural disasters (11–13) which describes vulnerable populations within a crisis situation, whereas the current reviewed focused on young populations in education, from the wider LMIC community. The LMIC context can increase the risk for mental health problems (particularly for youth with pre-existing psychosocial disadvantage). Further, schools can be useful contexts for detecting and supporting mental health problems in young people and serve as a way to homogenize the studies scoped (7, 14–16) and differ from more independent, older university students.

Mental health outcomes in young people arise from the interaction between individual psychological factors and the wider environmental resources available across multiple systems, as outlined in the framework of the socio-ecological model of resilience (17). This interplay - spanning individual, relational, community, and societal levels - is culturally shaped and supported by access to contextually appropriate resources. It is formed through innovations, co-production and expertise held in LMICs (18) but as yet the relationships between these variables remains elusive.

The rationale for this scoping review stems from a dispersed evidence base; the research on mental health problems and associated psychological factors for young people in LMICs offers a limited synthesis of findings to date. Coupled with an under-representation of LMIC contexts in the literature of young people’s mental health, associated psychological factors in global youth mental health remain under-researched (6).

This review will map the existing literature on mental health problems and associated psychological factors among young people in LMICs, including measurement tools, assessed outcomes and populations, and locations researched. By synthesizing evidence on what could constitute risk and protective factors in youth mental health in LMICs, the review can inform contextually relevant intervention approaches designed to promote mental health among young people in resource-constrained settings.

### 1.1 Aims and objectives

The overarching aims of this scoping review are the following:

1. To identify how young people’s mental health problems are measured and how they present in low socio-economic contexts.
2. To describe the psychological factors associated with young people’s mental health problems in LMIC countries.

Specific questions will address:

1. The mental health problems assessed in the research and the measures used to index them.
2. The psychological factors assessed alongside mental health problems, including resilience, self-esteem, self-efficacy, and coping; and the measures used to assess these.
3. The countries and time periods in which the studies have been conducted.
4. The composition of the samples used in the studies that assessed mental health problems and psychological factors, including age, gender and other characteristics.

## 2 Methods

This is a scoping review aimed at mapping evidence (measures, constructs, contexts), not assessing effectiveness or causality. It adopted the first five parts of the six-stage scoping review methodological framework recommended by Arksey and O’Malley (19): identifying the research question; identification of the relevant studies; study selection; charting the data; collating/summarizing/reporting the results. The framework was used as a step-by-step guide to create a narrative synthesis of the studies with flexibility given the heterogenous nature, geographical and cultural spread of the locations, and diverse methodology involved. We reviewed the process using the Preferred Reporting Items for Systematic Reviews and Meta-analysis Protocols for Scoping Reviews (PRISMA-ScR) checklist (20). The protocol was developed by the research team (TM, EN, AS), who have expertise in working with young people with mental disorders or distress, including those living in LMICs.

### 2.1 Stage 1: identifying the review questions

This scoping review maps the literature on the psychological factors found to be associated with mental health in school-attending youth in LMICs.

Following a search of key literature and discussion within the research team, it was found that no existing or scheduled reviews had been published (Cochrane Library, Campbell Collaboration, International Prospective Register of Systematic Reviews were searched) on the topic of psychological factors related to mental health problems in young people living in LMICs.

Inclusion criteria were defined in accordance with Population Concept Context in order to focus the search using the terms below:

- Population – School/College attending young people (up to 22 years of age)
- Concept: Mental health problems, resilience, self-efficacy, self-esteem, coping and other psychosocial mechanisms (to be scoped in the process)
- Context – LMICs

Table 1 defines the *a priori* inclusion/exclusion criteria applied during title/abstract and full-text screening.

**Table 1:** Eligibility criteria.

Studies were included if they:
Included a clear and defined measure of mental disorder The research assessed one or more clearly defined psychological variables Were published between inception of the database and 30th September 2025 Were published in English, in peer-reviewed journals or doctoral theses Assessed a formal area of mental health problem including screening measures of psychological distress Were a quantitative, qualitative or mixed-methods study Formally measured a psychological factor
Studies were excluded if they:
Referred to contexts in which populations were recruited following a natural disaster or war Recruited university attending students only Included samples with <51% of participants recruited within school Included a psychological factor not clearly operationalized but based on an interpretation of other data (e.g., resilience based on school attendance, or parental education level)

### 2.2 Stage 2: identification of relevant studies

A literature review was conducted using Medline, EMBASE and PsycINFO databases. All of the literature was published between 1970 to September 30th, 2025. The search strategy consisted of headings, keywords, and other related terms for the concept of psychological constructs, mental disorder presentations and LMICs. A detailed search strategy plan for each database is outlined in the see Supplementary material 1. The results were extracted to EndNote, duplicate results removed and then exported into Google Sheets for screening. This process was completed by one author (TM).

### 2.3 Stage 3: study selection

**Figure 1:**
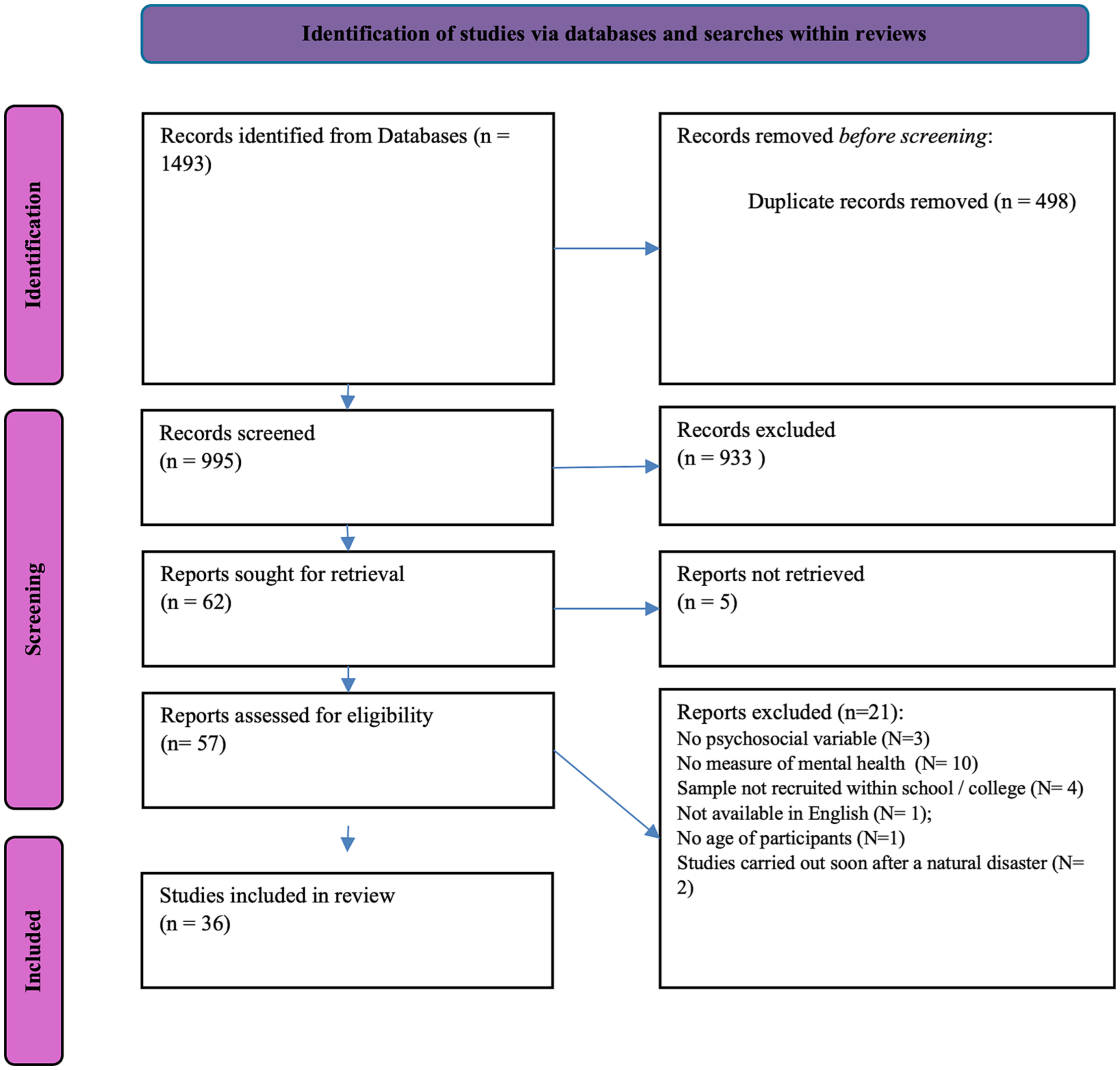
PRISMA diagram.

All articles were initially screened to meet the eligibility criteria by author (TM), then 10% were independently reviewed by author (AS), on the basis of their title and abstract, followed by the full text. Studies in which > 50% of the participants were recruited in school were included and where the student population was 22 years of age and under. No formal agreement metrics were used. Concerns about application of the criteria were discussed and agreed throughout the study selection process (TM, EN, AS). The PRISMA diagram which describes the searches and study selection process is depicted in Figure 1.

### 2.4 Stages 4 and 5: data extraction and charting the data

Articles that passed the screening process then underwent full interrogation. An abstraction template was developed by reading through the articles and is included in the protocol (21). The selected studies, after meeting the eligibility criteria, are presented in Table 2. The information consisted of bibliographical information (title; authors and year of publication; population; study design, country, aim; findings). Data were reviewed to identify key focus areas for interrogation and then summarized. Further details can be found in the published scoping review protocol (21).

**Table 2:** Details of all included studies.

First author and reference	Study type	Country	Sample size (N) and age	Aim	Findings
Ruangkanchanasetr et al. (35)	Cross-sectional	Thailand (Bangkok)	2,311 students (12–18 years)	To identify the prevalence of risk behaviors and related factors	High prevalence of risk behaviors (e.g., substance abuse, suicidal tendencies, injury) found; risk factors included poor self-esteem, poor school performance, and family factors
Habib and Seif El Din (53)	Intervention Study	Egypt (Alexandria)	334 (12–14 years) (*n* = 17 completed the intervention)	To evaluate the effectiveness of a Cognitive Behavior Therapy (CBT) program in reducing depressive symptoms	The CBT intervention resulted in significant improvement in self-esteem and reduction in depression symptoms 3 months after the completion of a 9-session program
Zhang et al. (43)	Cross-sectional	China (Shandong Province)	927 (14–15 years)	To investigate and compare the mental health problems and coping styles of adolescents in urban and rural areas and associations with socioeconomic status	Students in rural low-SES areas (especially females) had more mental health problems; maladaptive coping (venting/fantasizing) related to poorer mental health
He et al. (38)	Cross-sectional	China (Rural Hubei)	875 590 were ‘LBC’ (9–12 years)	To explore the risk of depression in Left-behind children and identify associated factors such as SES and social support	Left-behind children had a significantly higher risk of depression; low social support and low SES were associated with increased depression risk
Cortina et al. (36)	Prevalence Study	South Africa (Rural, Limpopo Province)	1,025 children (aged 10–12)	To determine the prevalence of mental health problems (emotional, conduct, and hyperactivity symptoms) in a rural, setting, and to identify associated risk factors, including perception/ connectedness to school	Teachers identified high levels of behavioral, conduct and emotional problems; second-generation refugee status and large household size were risk factors
Nik-Azin et al. (33)	Psychometric Validation Study	Iran (Yazd City)	551 students (11–19 years)	The Persian version of the Kidscreen-52 questionnaire was validated with mental health measures (anxiety, depression) and self-esteem	The Kidscreen-52 showed good internal consistency and acceptable construct validity; significant associations were found between self esteem and the KIDSCREEN-52 dimensions
Vawda (49)	Prevalence Study	South Africa (Durban)	222 (13–14 years)	To investigate the prevalence of suicidal behavior and associated psychological factors (social support, coping)	Low levels of self-esteem and perceived social support were associated with higher levels of suicidal behavior; alcohol use, depression, perceived stress, friends’ suicidal thoughts or ideation were risk factors for suicidal behavior
Leventhal et al. (26)	RCT	India (Rural Bihar)	2,308 rural females (12–14 years)	To evaluate the effectiveness of a 5-month school-based Resilience Curriculum (RC) program	The RCT showed significant improvements in measures of emotional resilience, self-efficacy, social–emotional assets, and wellbeing compared to controls. No effect on depression was detected. There was a small, negative effect of the intervention on anxiety.
Harrison et al. (32)	Cross-sectional	Jamaica (Metropolis)	524 (mean 14.9 years)	To determine the prevalence of Disordered Eating Behaviors (DEBs) and identify associated risk and protective factors, including self-esteem and mood	Risk factors for DEBs were: being female, history of sexual abuse and higher negative affect scores. A protective factor for participants was living with their father.
Satyanarayana et al. (27)	Cross-sectional	India (urban setting)	452 females (16–19 years)	To examine the associations among gender disadvantage, psychological distress, and resilience	A negative relationship was found between resilience and distress. Gender disadvantage (gender discrimination, violence and sexual harassment and barriers to personal growth) was associated with moderate and severe distress.
McMullen et al (25),	RCT	Uganda	620 with 170 reported post-intervention data (13–18 years)	To evaluate if a school-based, teacher-led life skills intervention was effective in increasing self-efficacy, prosocial behavior and a sense of connectedness and reducing internalizing problems	There was a significant increase in self-efficacy and connectedness and reduction in internalizing problems (depression and anxiety symptoms) following completion of the intervention compared to the control group.
Gardner and Lambert (52)	Cross-sectional	Jamaica	334 (10–18 years)	To examine the interacting effects of Trait-Emotional Intelligence (TEI) on the associations between self-esteem and depressive symptoms	Older adolescents had greater depressive symptoms and lower self-esteem. TEI’s buffered the association between self-esteem and depressive symptoms
Nsabimana et al. (24)	Cross-sectional	Rwanda	178 (Aged 9–16)	To investigate if institutionalization negatively impacts children’s psychological adjustment (externalizing/internalizing problems and self-esteem)	Institutionalized children had significantly more externalizing behavior problems. Self-esteem of non-orphans in families was highest, highlighting the protective role of family
Mwakanyamale et al. (54)	Cross-sectional	Tanzania	1,000 (13–21 years)	To describe the relationship between childhood psychological maltreatment, self-esteem, psychological distress, depression and anxiety	Psychological maltreatment positively correlated with psychological distress and negatively correlated with self-esteem
Pandey et al. (47)	Prevalence study	Nepal	6,531 students (12–17 years)	To estimate the prevalence of suicidal ideation and identify the associated factors	Anxiety, loneliness, and female gender were risk factors for both suicidal ideation and attempts; having 3 or more close friends was a protective factor against suicidal attempt
Rana et al. (34)	Prevalence study	India (Chandigarh, North India)	667 (12-15 years)	To estimate the prevalence and correlates of bullying	Bully-victims had the highest mental health problems. Predictors of bullying included: being male, having ‘abnormal’ emotions, and poor peer relations. Self-esteem was not found to correlate with bullying.
Arora et al. (28)	Qualitative Study	Guyana	40 (12–17 years) (and 17 adults)	To explore youth and adult stakeholders’ perspectives on risk and protective factors for suicide	Risk factors included: pressure and expectations, adults’ negative responses to youth, limited coping, and exposure to suicide; protective factors included: positive social support and involvement in community activities
El-Abbassy et al. (45)	Intervention	Egypt 54% rural, 46% urban	80 (13–15 years)	To examine the efficacy of a Positive Psychology Intervention (PPI) in promoting mental health, life satisfaction, self-esteem, optimism and happiness	The PPI led to increases in self-efficacy, self-esteem, optimism, life satisfaction, and happiness scores from pre-intervention to post-intervention
Garbett et al. (31)	Pilot RCT	Urban India	166 (11 years)	To evaluate a culturally adapted school-based body image intervention “Dove Confident Me”	The intervention demonstrated high acceptability and preliminary efficacy and significant improvements in body esteem and positive affect scores; no significant effects were found on internalization, life engagement, eating pathology, self-esteem or negative affect
Addy et al. (44)	Mixed-Methods Study	Ghana (rural and peri-urban)	Quantitive study - 405 (12–20 years) Qual – 53 participants including 35 students	To investigate mental health problems, coping strategies, and the student’s support systems	Risk factors included being female, bullying, domestic violence, substance abuse, and academic pressure, coping involving isolation, substance use, and spiritual help; school counseling units were often ineffective
Avanci et al. (29)	Prevalence study	Brazil (São Gonçalo, Rio de Janeiro) Urban dwellers	862 (13–19 years)	To determine the prevalence of Post Traumatic Stress Disorder symptoms in trauma-exposed students and investigate associated factors (sociodemographic, individual, and family)	Significant risk factors included social functional impairment and violence. Resilience and social support were not significantly associated with the presence of PTSD symptoms.
Wang et al. (42)	Cross-sectional	Rural China (Gansu province)	2,989 (7–15 years)	To examine the relationship between primary caregivers’ mental health and school-aged children’s outcomes (mental health, resilience, academic performance)	Caregiver mental health problems were negatively correlated with all student outcomes (student mental health, resilience, academic performance)
Berry et al. (30)	Quasi-experimental Trial	South Africa (Cape Town Metropolitan area)	113 (12–22 years)	To assess outcome of depression, resilience and positive parenting in young people who participated in a 20 week adolescent parenting program which included biological and nonbiological parents	Positive parenting and resilience improved in both groups. Depression rates increased for non-biological parents in the intervention group. High depression risk was associated with smaller improvements in supportive parenting.
(46)	Short-term Longitudinal	Peru (throughout the nation)	1,334 (11–17 years)	To investigate whether social-ecological resilience (personal resources and caregiver relationship) related to changes in internalizing symptoms (anxiety and depression) during the COVID-19 lockdown	Adolescents with higher levels of personal, caregiver, and overall resilience had lower levels of anxiety and depressive symptoms; resilience moderated the change in anxiety symptoms over time
She et al. (48)	RCT	China (Shanghai, rural and urban)	653 (9 to 16 years)	To test the feasibility and effectiveness of a volunteer-led, 8-week mindfulness intervention	No statistically significant differences were found for mindfulness, resilience, anxiety, or depression outcomes in the intervention vs. control groups
Alshammari (57)	Cross-sectional	Jordan (Irbid governorate)	2,741 (13–18 years)	To explore the risk and protective factors related to health behaviors (tobacco, diet) and mental health and psychological variables (depression, life satisfaction, self-esteem)	The importance of social support (from family, friends, and significant others) was a protective factor against depressive symptoms and higher self-esteem and life satisfaction
Edet et al. (37)	Cross-sectional	Nigeria (Calabar)	332 (11–20 years)	To assess the relative importance of social support and family affluence in depression and self-esteem	Social support was of greater importance than family affluence; respondents with low social support had significantly higher depression and lower self-esteem scores, regardless of their family affluence. Social support and female gender predicted depression; social support and age was positively associated with self-esteem
Lewis-Smith et al. (39)	RCT	India (Delhi)	568 11–14 years)	To evaluate the efficacy of a school-based mixed-gender body image intervention (“Dove Confident Me”)	The intervention led to significant improvements in body image at post-intervention and 3-month follow-up. Significant improvements were found for internalization, life disengagement, disordered eating, self-esteem, and negative affect.
Tran et al. (40)	RCT	Vietnam (Hanoi)	1,084 (15–16 years)	To evaluate the effects of a universal school-based mental health promotion program, ‘Happy House’	At 2 weeks post-intervention, the program participants showed significant reductions in depressive symptoms and higher psychological well-being. Coping and self-efficacy were higher in the intervention group at 2-week and 6-month follow-up. The effect on depressive symptoms was no longer significant at 6 months.
Cherewick et al. (56)	Cross-sectional	India (Darjeeling) urban, peri-urban, and rural	274 (10–14 years)	To analyze the relationship between autistic traits and internalizing symptoms among early adolescents and examine the moderating effect of self-efficacy	Higher internalizing symptoms were associated with higher autistic traits; self-efficacy (academic, social, and emotional dimensions) moderated the relationship
Coetzee et al. (50)	Pilot Test	South Africa (Western Cape)	222 (9–11 years)	To determine the feasibility and acceptability of ‘4 Steps To My Future’ (4STMF), a universally delivered classroom-based mental health program	The program was acceptable and feasible. Exploratory outcomes showed significant pre-post improvements in self-esteem and emotion regulation.
Ward-Smith et al. (55)	Cross-sectional	South Africa (Western Cape)	733 (15–18 years)	To investigate associations between depression and anxiety symptoms and Emotion Regulation (ER)	Female gender, higher depressive symptoms, anxiety, PTSD symptoms and risky-alcohol use were significantly associated with poorer ER, whereas self-esteem was significantly associated with better ER
Chen et al. (51)	Cross-sectional	China (Guangdong Province)	686 (11–16 years)	To assess whether shift-and-persist strategies were associated with depressive symptoms and have Socio Economic Status (SES) may be related	Use of more frequent shift-and-persist strategies was associated with fewer depressive symptoms, with stronger effects among those with lower SES.
Wan et al. (41)	Cross-sectional	China (Rural, 8 provinces)	4,708 (12 years)	To examine whether left-behind families and gender inequality places children at higher risk of Adverse Childhood Experiences (ACEs) and depressive symptoms	There was a positive association between the number of ACEs reported and symptoms of depression and low self-esteem
Zook et al. (23)	Intervention Study	Democratic Republic of Congo	483 females (10–14 years)	To assess whether a 12 week music therapy Healing in Harmony (HiH) program improved mental health and school attendance	The HiH program was associated with improvements in depression and higher self-esteem post-intervention and at 17 month follow up
Fine et al. (22)	Cross-sectional qualitative Study	9 LIMCs: Chile, China, Democratic Republic of the Congo, Egypt, Indonesia, Jamaica, Jordan, Kenya, Malawi; 4 HICs: Belgium, Sweden, Switzerland, United States	71 (12–19 years)	To understand adolescents’ perspectives on mental health challenges, risk and protective factors, and coping strategies in multi-country settings	In LMICs and HICs young people emphasized coping, and social and contextual factors (friends, family school, poverty, violence) to be associated with mental health problems and barriers to help-seeking.

### 2.5 Stage 6: collating, summarizing and reporting the results

The original articles were re-examined twice to ensure that the relevant details were accurately captured. The summaries and reporting plans were then integrated into a final draft by TM and AS then discussed with the other authors.

#### 2.5.1 Ethics and dissemination

Ethical approval was not required as this study did not directly involve data collection from human participants.

## 3 Results

The selected studies, after meeting the eligibility criteria, are presented in Table 2. The information consisted of bibliographical information (title; authors and year of publication; population; study design, country, aim; findings). Data were reviewed to identify key focus areas for interrogation and then summarized. Further details can be found in the published scoping review protocol (21).

A total of 36 studies were identified in 22 LMICs. Four of the 36 studies were reported in LICs (Malawi) (22), Democratic Republic of Congo (22, 23), Rwanda (24) and Uganda (25)) and 32 were reported in MICs (Lower-Middle-Income Countries Egypt, Indonesia, Kenya, Ghana, India, Nepal, Nigeria, and Vietnam) and Upper-Middle-Income Countries (Brazil, China, Guyana, Iran, Jamaica, Jordan, Peru, South Africa and Thailand). One study (22) included data from multiple countries (of which 9/13 were LMICs at the time the study was carried out: Chile, China, Democratic Republic of the Congo, Egypt, Indonesia, Jamaica, Jordan, Kenya, and Malawi).

### 3.1 Participants

The age of participants ranged from 7 to 22 years. Thirty-five of the studies included males and females, and 3 reported on females only (23, 26, 27). Within the quantitative studies, the samples were varied in size, from 80 to 6,531 participants, whereas the qualitative studies included 41 (28) and 71 students (22). The studies reported a mix of demographics from urban settings (25, 27, 29–35), rural settings (24, 36–43) and a mix of urban and rural settings (26, 41, 44–49). Eleven studies did not specify the exact region of where the sample was recruited.

### 3.2 Education contexts

Where reported, the number of schools included in the studies varied from one school (28, 45) to 73 (29), 74 (47) and 80 (37) schools. The samples were recruited from a mix of primary (24, 36, 38, 45, 48, 50), secondary (25, 26, 28–35, 37, 39, 40, 43–47, 49, 51–55); and a combination of primary and secondary schools (23, 41, 42). Cherewick et al. (56) did not report on the type of school included in the study while Satyanarayana et al. (27) studied college participants. Several studies specified that their samples were recruited in public/government funded schools (26, 39, 40, 44, 49, 50) or private schools (28, 31, 46, 56). Other studies denoted a combination of government and private schools (29, 34, 37, 48, 57). Twenty studies did not report on the type of school included (22, 24, 25, 27, 30, 32, 33, 35, 36, 38, 41–43, 45, 47, 51–55). Zook et al. (23) noted that the schools included were funded through a large charity in the Democratic Republic of Congo.

**Figure 2:**
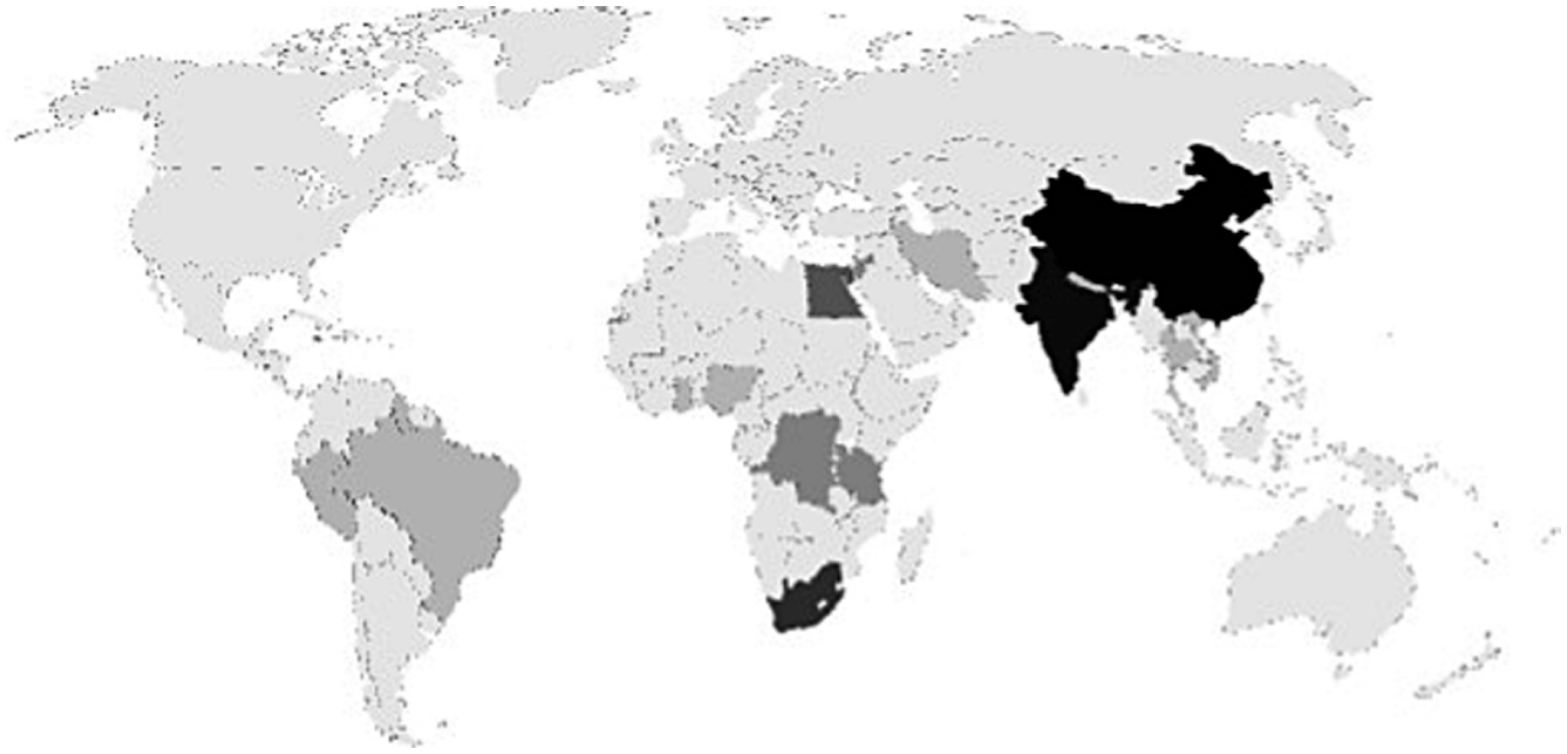
Map of countries showing included studies. The license for map is: https://commons.wikimedia.org/wiki/File:World_Locator.svg.

The countries in which the studies were carried out across three continents are shown in Figure 2. The majority of studies were from Africa (9/36 - South Africa, Uganda, The Gambia, Tanzania, Rwanda, Egypt, Ghana, Nigeria, Democratic Republic of Congo).

**Figure 3:**
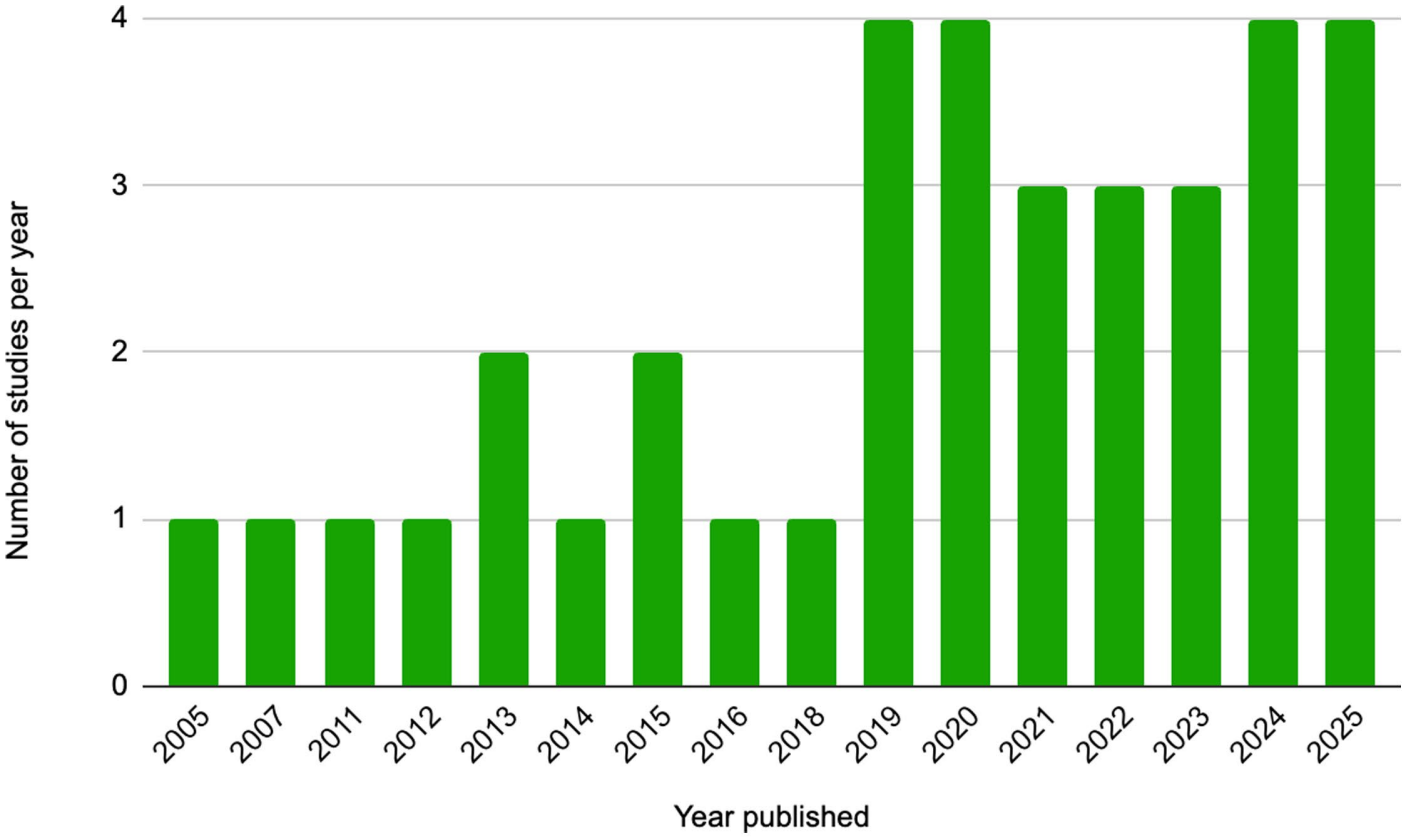
Timeline of studies published by year between 2005 and 2025.

Figure 3 shows a timeline of the studies published to date. The first study identified was published in 2005 with the majority of articles having been published since 2019.

#### 3.2.1 How mental health was measured

Several areas of mental health problems were measured. The most common measures assessed symptoms of depression (23, 26, 30, 35, 36, 38, 40–42, 46, 48–53, 55, 57); anxiety (23, 26, 36, 42, 46–48, 50, 55); trauma (29, 36, 54, 55); suicidality (28, 35, 47, 49); disordered eating (31, 32, 39), and aggression (49). There were also measures of psychological distress (25, 26, 54), negative/risky behavior (35) and general mental health screening (24, 31, 33, 34, 39, 43–45, 50). The Center for Epidemiologic Studies Depression Scale (CESD) (58) was the most commonly used inventory to assess mental health problems across the included studies (40, 48, 51, 55, 57).

#### 3.2.2 Psychological factors associated with mental health

A range of psychological factors were also investigated in the included studies in terms of their association with mental health problems. Most studies assessed more than one area of psychological function. The most commonly assessed factors were self-esteem (23, 24, 31–35, 37, 39, 45, 49, 50, 52–55, 57), resilience (26, 29, 30, 42), coping (22, 43, 44, 46, 51), social support (22, 28, 29, 37, 38, 41, 47, 49, 55, 57), self-efficacy (25, 26, 40, 45, 56). School connectedness (36); emotional intelligence and emotional regulation (50, 55), optimism (45), life satisfaction (33, 57), personal control / mastery (49), connectedness (25), trait emotional intelligence (52), and Mindfulness (48) were also measured.

Two studies used versions of the same measures of resilience, i.e., the Connor-Davidson Resilience Scale-10 (28, 42) and a notable number of studies used versions of the Rosenberg Self-Esteem Scale (RSES) (23, 31, 32, 34, 37, 39, 45, 49, 50, 52, 54, 55, 57, 140). Two studies used the Child and Youth Resilience Measure (CYRM-28) (43, 46, 59).

In two studies (26, 42), an adult validated measure of resilience was used (Connor–Davidson Resilience Scale) (60) and three studies (37, 40, 49) drew on the Beck Depression Inventory (61) in young people.

The standardized measures used to assess mental health problems and associated psychological factors are described in Table 3.

**Table 3:** Measures used to assess mental health and associated psychological constructs.

Mental health measures	Psychological construct measures
Depression and suicidality	Resilience
Beck’s Depression Inventory (BDI) (61) Beck’s Hopelessness Scale (BHS) (85) Birleson Depression Self-Rating Scale for Children (86) Center for Epidemiologic Studies Depression Scale (CESD-10) Center for Epidemiologic Studies Depression Scale for Children (CES-DC) (62) Child Depression Inventory; Arabic and Chinese versions (CDI) (87, 88, 141) Depression, Anxiety and Stress Scale-Short Form (88) DSM-5 Depression scale (89) Kutcher Adolescent Depression Scale (KADS) (90)	Child and Youth Resilience Measure (CYRM-28) (59) Child and Youth Resilience Measure Revised (CYRM-R) (67) Connor-Davidson Resilience Scale-10 (60) Resilience Scale (69)
Anxiety	Self-esteem and self-efficacy
General Anxiety Disorder-7 (GAD-7) (91) Hopkins Symptom Checklist (HSCL) (92) Multidimensional Anxiety Scale for Children Chinese version (MASC) (93) Revised Child Anxiety and Depression Scale (RCADS-30) (94) Youth Self Report (YSR) (95)	Coping Self-Efficacy Scale (CSES) (96) Coopersmith Self-Esteem Inventory (CSEI) (65) General Self-Efficacy Scale (GSE) (144) Rosenberg Self-Esteem Scale (RSES) (140) Rosenberg Self-Esteem Scale Short Form (68)
General mental health	Social connectedness and perceived social support
African Youth Psychosocial Assessment Instrument (97) Brief symptom Inventory (98) Child Behavior Checklist (99) General Health Questionnaire (GHQ-28) (100) Global School-based Students Health Survey (GSHS) questionnaire Nepali version KIDSCREEN-52 Self-report (101) Mental Health Continuum Short Form (MHC-SF) (102) Mental Health Inventory for Adolescents (MHI-A) (103) Pediatric Quality of Life Inventory (PedsQL) (143) Patient Health Questionnaire (PHQ) (104) Personal Well-being Index- School Children (PWI-SC) (105) Positive and Negative Affect Schedule for Children (106) Strengths and Difficulties Questionnaire (SDQ) (63)	Child and Adolescent Social Support Scale (107) Hemingway Measure of Adolescent Connectedness (MAC-5) (108) Multidimensional Scale of Perceived Social Support (MSPSS) (109) Multidimensional Scale of Perceived Social Support Chinese version (MSPSS) (139) Oslo Social Support Scale (OSS) (110) Perceived Social Support Scales for Family and Friends (111) Self-Efficacy Questionnaire for Children (SEQ) (112) Social Support Battery (113)
Trauma	Coping
Childhood Trauma Questionnaire (CTQ) (114) Child and Adolescent Trauma Survey (CATS) (115) Trauma Symptom Checklist for Children –Alternate form (TSCC-A) (116) UCLA Post-Traumatic Stress Disorder Reaction Index (117)	Coping Style Scale of Middle School Students (CSSMSS) (66) Shift and Persist Questionnaire (64)
Disordered eating and body image	Life satisfaction
Body Esteem Scale for Adolescents and Adults (118) Body Image Life Disengagement Questionnaire (119) Eating Attitudes Test (EAT-26) (120) Eating-Disorder Examination Questionnaire (121)	Multidimensional Students’ Life Satisfaction Scale (MSLSS) (122) Satisfaction with Life Scale (SWLS) (123)
Psychological distress	Emotional regulation
Kessler psychological distress scale (K10) (124) Patient-Reported Outcomes Measurement Information System Short Form (PROMIS) (125) Perceived Stress Scale (126) Thai Youth Risk Behavior Survey Form Youth Self Report (YSR) (95)	Emotional Regulation Questionnaire for Children and Adolescents (ERQ-CA) (127) Self-Rated Difficulties of Emotion Regulation Scale-16 (DERS-15) (128)
Other	Other
Aggression Scale (129) Autism Spectrum Quotient (AQ) (130) Brief Impairment Scale (BIS) (131) Olweus Bully/Victim Questionnaire (132)	Life Orientation Test-Revised (LOT-R) (133) Mastery Scale (134) Mindfulness Attention Awareness Scale Chinese version (MAAS) (135) Oxford Happiness Questionnaire (OHQ) (136) Peace Zone Questionnaire (142) Schutte Emotional Intelligence Scale (137)

There was a broad range of inventories used; several studies reported adapting and piloting existing measures locally (23, 25, 29, 35, 36, 38, 42, 48, 51, 52) while some studies reported that measures had been translated and backtranslated from English to the local language (31, 33, 39, 43, 46, 48).

The majority of the studies (*n* = 35) were quantitative, two were qualitative (22, 28), and one included mixed methods (45). Most of the studies were cross-sectional, several described intervention programs (23, 30, 31, 45, 50, 53), several included randomized control trials focused on mental health and psychological variables as outcome measures (26, 39, 40, 48). One study described the validation of the Persian version of the Kidscreen-52 questionnaire (33). There were five prevalence studies of mental health problems (29, 34, 36, 47, 49) and two longitudinal studies that assessed change in mental health problems and psychological variables over time (46, 51).

## 4 Discussion

### 4.1 Main findings

This scoping review identified 36 empirical studies in young people which were reported in a limited number of LICs, with most studies (>30%) having been carried out in China and India since 2019.

The most common mental health problem measured in the studies was depression, assessed with the Center for Epidemiologic Studies Depression Scale (58) or a briefer version of the scale adapted for children (Center for Epidemiologic Studies Depression Scale for Children) (62). Other common mental health problems also measured through standardized inventories included anxiety (23, 26, 36, 42, 46, 47, 50, 55, 56), trauma (29, 36, 54, 55) and externalizing conditions, such as hyperactivity (24, 44, 49). Brief general mental health screening was commonly used, such as the Strengths and Difficulties Questionnaire (63). Eating disorder was included in studies of youth living in LMICs (31, 32, 39).

The measures used to assess the various psychological factors were wide ranging. The most commonly measured psychological factor associated with youth mental health problems was self-esteem, which was most frequently assessed using the Rosenberg Self-Esteem Scale (140). Notably, self-efficacy, resilience and coping were assessed through a diverse range of measures developed in HICs; (59, 60, 64–69, 140) (Table 3). Perceived social support (22, 28, 29, 37, 57) was widely assessed alongside positive psychology and cognitive factors such as school connectedness/school perception (25, 36), life satisfaction (57), and optimism (45).

Most interventions were reported to be linked to enhanced resilience (26), self-esteem and optimism (45, 50), body image (39), and coping and mood (40). One study adapted a music intervention which was associated with improvements in measures of mental health problems and self-esteem (23). Notably, both Garbett et al. (31) and Lewis-Smith et al. (39) used a similar intervention which was related to enhanced body-image, called “Dove Confident Me” in India and had different results, with favorable results in the latter, larger study. However, some interventions did not result in a different outcome on psychological (31) or mental health measures from a comparison group (48).

This scoping review showed that while mental health and associated psychological variables were responsive to change post intervention, the relationship between the mental health and the various identified psychological factors associated with mental health was not consistently explored in intervention trials or cross-sectional studies. However, in several studies, self-esteem was found to correlate with lower psychological distress and better mental health outcomes, such as reduced rates of depression (23, 37, 53), anxiety (23) and suicidality (49). Effective coping styles were also noted to be associated with fewer mental health problems, particularly shift-and-persist strategies (50) while use of maladaptive coping such as venting and fantasizing (43); isolation and substance use was also a risk factor for increased mental health problems, such as hyperactivity, conduct problems, suicidality and depressive symptoms (44). Emotion-focused coping was associated with increased reports of anxiety and depression (55).

Cherewick et al. (56) found that self-efficacy moderated the relationship between high internalizing symptoms and autistic traits in a group of Indian young people. In addition, Guazzelli Williamson et al. (46) showed that in a group of Peruvian students, those with higher levels of resilience reported lower levels of anxiety and that over the course of several weeks during the COVID-19 pandemic, resilience moderated the change in their anxiety symptoms. This pattern of findings could suggest that focusing research on such psychological variables may provide valuable insights for the assessment and management of mental health in young people living in LMICs. Although, it should also be emphasized that the current findings are context-specific, heterogeneous, and not suitable for synthesis or generalization.

Importantly, while a broad age range of young participants was used to evaluate this literature, many studies described a fairly narrow band of developmental stage of 1–2 years (31, 39, 49). However, given the heterogeneity of the studies and their findings, it makes it difficult to compare findings across ages, and this was not a focus within the studies themselves, although adolescence is identified as a greater time of mental health vulnerability than childhood (70).

#### 4.1.1 Protective and risk factors

The review yielded protective and risk factors associated with youth mental health problems which could be mapped to a broader concept of resilience, as depicted in the socio-ecological theoretical framework (17).

Higher rates of trait-emotional intelligence were linked to improved self-esteem and lower depressive symptoms (52). Stronger social support was associated with better mental health outcomes (28, 32, 37, 47, 55, 57). Having more than 3 friends (47) and involvement in community activities (28, 37, 57) outperformed other factors such as family affluence (37). In Jamaica, living with a father was associated with fewer eating disorder symptoms (32).

Associated risk factors for mental health problems included psychological maltreatment (54), exposure to violence (22, 29), institutionalization (24), bullying, substance abuse, and academic pressure (44), mental health problems in primary caregivers (42), negative life events and emotional problems (51), gender disadvantage (27), Adverse Childhood Experiences (41) and poverty (22), particularly in ‘Left Behind Children’ with low levels of social support (38).

Several studies showed that young females are more at risk of mental health problems than young males, a pattern that has also been reported in HICs (71). It is likely that this persistent vulnerability in girls and young women may underlie the reason why three studies in this review had invested resources in research focused on females alone (23, 26, 27). Consideration of the factors identified suggests that cross-cultural environmental factors are likely to contribute to the variation observed within and between studies. Further cross-cultural research would address issues as highlighted by the World Health Organization to employ gender mainstreaming in worldwide research of youth mental health (72).

Noteworthily, all studies in the review utilized a binary approach to gender, potentially omitting key stressors implicated in gender-diverse young people’s mental health in LMICs, as reported in HICs (73, 74) and LICs (5). Hyde et al. (75) suggest that within psychological and mental health research “the tendency to view gender/sex as a meaningful, binary category is culturally determined and malleable”. Limitations of using this strict categorization could serve to exclude particularly vulnerable groups and limit generalizability of findings. The acceptance of mental health as a human right for all vulnerable groups including transgender people has been highlighted as a priority within global mental health (76). Furthermore, non-binary adults from MICs such as Brazil (77) and Iran (78) have been shown to be vulnerable to mental health problems.

Mental health problems in LMICs arise from complex, multi-factorial processes, as highlighted by the socio-ecological framework of resilience (17). While this review focuses on individual psychological factors, the framework emphasizes that these factors are shaped by community, societal, relational, and environmental contexts rather than existing independently (79). Therefore, the causal pathways between psychological and social community factors in the mental health of young people in LMICs appears to be complex, bidirectional and as yet unclear.

### 4.2 Limitations

This review has a number of limitations. Most of the literature screening was conducted by one reviewer with partial cross-checking, consistent with scoping review practice. The lack of inclusion of non-English language papers led to the omission of literature produced from non-English speaking countries. As the review focused on studies which collected data in LMICs, it did not capture data from resource constrained groups within HICs, such as refugees from LMICs who moved to HICs or indigenous communities. Similarly, as the study samples were education-attending, the conclusions drawn are restricted to these populations and not to other likely vulnerable groups, such as homeless children or those not in education due to illness or economic adversity. Although almost all the samples included studies with school-recruited young people, some studies included a small number of participants who were recruited from other contexts, such as orphanages (24, 35) and other communities (35).

A significant limitation relates to the conceptual and methodological difficulty of employing measures of mental health problems and psychological variables developed in HICs in LMIC populations. The lack of cultural validity and measurement equivalence to capture the risks and protective factors has been presented as a barrier to research in these regions (80, 81). Use of measurement developed beyond the culture it is used in could result in diagnostic distortion of either inflated or deflated detection which then might mean that intervention is not provided or assessed in youth who require it. However, there is evidence of adult studies from LICs which have developed culturally informed, locally developed and psychometrically robust instruments which should be held as a standard for future research in young people, with associated funding identified to facilitate it (81, 82). Notably, the implications of research which employs constructs developed in HICs, such as self-esteem and self-efficacy, risks reduced conceptual validity in LMICs and potential to overly focus on individualistic constructs which are self-oriented rather than relational or communal constructs. The growing appreciation of young people as ‘active agents’ in the co-design and implementation of mental health research in LMICs will likely help to improve research and its findings (83, 84).

Due to methodological and pragmatic constraints, another limitation was that the study selection process was conducted by one researcher primarily; while a second reviewer examined 10% of the studies, the robustness of this scoping review would be enhanced with a more stringent screening approach.

### 4.3 Implications for practice and future research

There are strengths in the review of these studies which could inform future research. While there was notable inconsistency in how mental health aspects were assessed, many of the reviewed studies used large sample sizes (24, 26, 32, 35, 44, 50), translation and back-translation of measures (31, 33, 39, 46), and made efforts to facilitate valid measure completion by reading the measures to participants (23, 45, 51). Such practices highlight important considerations for LMIC-oriented studies, particularly in ensuring robust sampling, consistent and culturally sensitive measurements, and accessible data collection.

## Data Availability

All data used in this scoping review are derived from publicly available published literature. The studies included in the review are cited within the manuscript. No new data were created or analysed in this study.
