# Supplementary material for: A scoping review of mental health problems and associated psychological factors pertinent to young people in education in low-and middle-income countries
Source: Front Public Health. 2026 Jun 18;14:1842137. doi: 10.3389/fpubh.2026.1842137 (PMC13322913; doi:10.3389/fpubh.2026.1842137)
Supplement: Supplementary file 1 [file Supplementary_File_1.docx]

# Supplementary material 1

# Electronic Search Strategy

## Sources and databases:

- Medline
- Embase
- APA Psychinfo

Limits applied:

| **Age group** | **Language** | **Publication type** | **Time limit** |
| --- | --- | --- | --- |
| 4-22 years | English | All | 1970 to date |

## Search strings

Ovid MEDLINE(R) ALL <1946 to September 29, 2025>

Date of search: 30/09/25

Results: 481

1 (adolescen* or teen* or young person* or young people or young adult* or child*).ti,ab,kf. 2219837

2 Adolescent/ 2355552

3 exp Child/ 2289615

4 Young Adult/ 1167766

5 1 or 2 or 3 or 4 4788235

6 (resilien* or self-efficacy or coping or self-esteem).ti,ab,kf. 240791

7 Resilience, Psychological/ 12167

8 self efficacy/ 27940

9 Coping Skills/ 893

10 6 or 7 or 8 or 9 250051

11 (mental health or mental wellbeing or mental well being or mental ill* or mental disorder* or psychiatric condition* or psychological distress or psychological stress* or depression or depressive symptom* or anxiety or traum* or DSM or ICD).ti,ab,kf. 1514032

12 Mental Health/ 74845

13 exp Mental Disorders/ 1567737

14 Stress, Psychological/ 144846

15 Psychological Distress/ 6470

16 Depression/ 174032

17 Anxiety/ 124342

18 Psychological Trauma/ 2291

19 "diagnostic and statistical manual of mental disorders"/ or "international classification of functioning, disability and health"/ 19698

20 11 or 12 or 13 or 14 or 15 or 16 or 17 or 18 or 19 2773998

21 ("low and middle income countr*" or LMIC* or low socio-economic or low socioeconomic or developing countr* or economically disadvantaged region* or under-resourced health system* or resource-limited health system* or resource limited health setting*).ti,ab,kf. 163278

22 Developing Countries/ 84580

23 21 or 22 199606

24 5 and 10 and 20 and 23 481

Embase Classic+Embase (Ovid) <1947 to 2025 September 26>

Date of search: 30/09/25

Results: 720

1 (adolescen* or teen* or young person* or young people or young adult* or child*).ti,ab,kf. 3089800

2 exp Adolescent/ 2159553

3 exp Child/ 3943535

4 Young Adult/ 652030

5 1 or 2 or 3 or 4 6099520

6 (resilien* or self-efficacy or coping or self-esteem).ti,ab,kf. 291409

7 exp Psychological Resilience/ 17336

8 exp Self Esteem/ 31780

9 Coping/ 86126

10 6 or 7 or 8 or 9 327847

11 (mental health or mental wellbeing or mental well being or mental ill* or mental disorder* or psychiatric condition* or psychological distress or psychological stress* or depression or depressive symptom* or anxiety or traum* or DSM or ICD).ti,ab,kf. 2163217

12 exp Mental Health/ 334409

13 exp Mental Disease/ 3383190

14 mental stress/ 114914

15 depression/ or adolescent depression/ 611702

16 anxiety/ 379736

17 psychotrauma/ 13516

18 exp "diagnostic and statistical manual of mental disorders"/ or "international classification of functioning, disability and health"/ 78239

19 11 or 12 or 13 or 14 or 15 or 16 or 17 or 18 4685117

20 ("low and middle income countr*" or LMIC* or low socio-economic or low socioeconomic or developing countr* or economically disadvantaged region* or under-resourced health system* or resource-limited health system* or resource limited health setting*).ti,ab,kf. 175670

21 Developing Country/ 107251

22 20 or 21 234990

23 5 and 10 and 19 and 22 720

24 limit 23 to yr="1970 -Current" 720

APA PsycInfo (Ovid) <1806 to September 2025 Week 3>

Date of search: 30/09/25

Results: 292

1 (adolescen* or teen* or young person* or young people or young adult* or child*).ti,ab,id. 1101088

2 early adolescence/ 3789

3 late adolescence/ 562

4 emerging adulthood/12113

5 1 or 2 or 3 or 4 1104359

6 (resilien* or self-efficacy or coping or self-esteem).ti,ab,id. 254220

7 "resilience (psychological)"/ 27800

8 self-efficacy/ 34936

9 exp coping behavior/ 60319

10 self-esteem/ 29782

11 6 or 7 or 8 or 9 or 10 263160

12 (mental health or mental wellbeing or mental well being or mental ill* or mental disorder* or psychiatric condition* or psychological distress or psychological stress* or depression or depressive symptom* or anxiety or traum* or DSM or ICD).ti,ab,id. 936584

13 exp mental health/ 112822

14 exp mental disorders/ 1130553

15 emotional well being/ 570

16 adolescent psychiatry/ 7192

17 psychological stress/ 10271

18 distress/ 34844

19 "depression (emotion)"/ 27630

20 psychiatric symptoms/ 21067

21 anxiety/ 88850

22 emotional trauma/ 16778

23 12 or 13 or 14 or 15 or 16 or 17 or 18 or 19 or 20 or 21 or 22 1632301

24 ("low and middle income countr*" or LMIC* or low socio-economic or low socioeconomic or developing countr* or economically disadvantaged region* or under-resourced health system* or resource-limited health system* or resource limited health setting*).ti,ab,id. 24906

25 developing countries/ 6851

26 24 or 25 26822

27 5 and 11 and 23 and 26 292

28 limit 27 to yr="1970 -Current”
